# Mitigating tradeoffs in plant breeding

**DOI:** 10.1016/j.isci.2021.102965

**Published:** 2021-08-10

**Authors:** Sangam Lal Dwivedi, Matthew Paul Reynolds, Rodomiro Ortiz

**Affiliations:** 1 Independent Researcher, Hyderabad 500016, Telangana, India; 2 International Maize and Wheat Improvement Centre, Mexico; 3 Swedish University of Agricultural Sciences (SLU), Alnarp, Sweden

**Keywords:** Biological sciences, Biotechnology, Plant biology, Plant biotechnology, Plant Genetics

## Abstract

Tradeoffs among plant traits help maintain relative fitness under unpredictable conditions and maximize reproductive success. However, modifying tradeoffs is a breeding challenge since many genes of minor effect are involved. The intensive crosstalk and fine-tuning between growth and defense responsive phytohormones via transcription factors optimizes growth, reproduction, and stress tolerance. There are regulating genes in grain crops that deploy diverse functions to overcome tradeoffs, e.g., miR-156-*IPA1* regulates crosstalk between growth and defense to achieve high disease resistance and yield, while *OsALDH2B1* loss of function causes imbalance among defense, growth, and reproduction in rice. *GNI-A1* regulates seed number and weight in wheat by suppressing distal florets and altering assimilate distribution of proximal seeds in spikelets. Knocking out ABA-induced transcription repressors (AITRs) enhances abiotic stress adaptation without fitness cost in *Arabidopsis*. Deploying *AITRs* homologs in grain crops may facilitate breeding. This knowledge suggests overcoming tradeoffs through breeding may expose new ones.

## Graphical abstract

**Figure:**
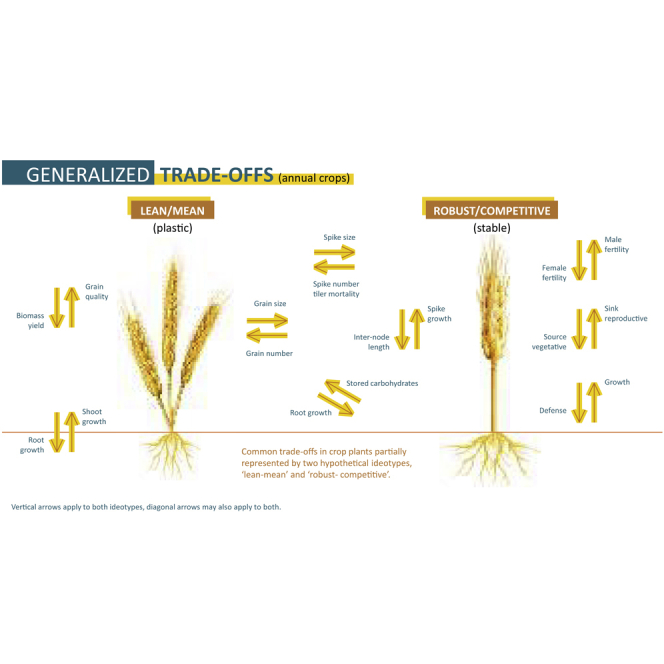

## Introduction

Today’s agriculture is facing unprecedented challenges to provide nutritious and safe food to a growing world population. The global population is projected to peak in 2064 at 9.73 billion (8.84–10.9) people and decline to 8.79 billion (6.83–11.8), and a shifting age structure, 2.37 billion (1.91–2.87) individuals older than 65 years and 1.70 billion (1.11–2.81) individuals younger than 20 years, globally in 2100. Continued trends in female education attainment and access to contraception will hasten declines in fertility and slow population growth (Vollset et al., 2020). The incorporation of ‘*Green Revolution*’ genes in newly bred cultivars, along with the use of inorganic fertilizers, irrigation, and farm mechanization led to multiple-fold increase in production of main staples such as maize, rice, soybean, and wheat. These grain crops produce nearly two-thirds of the calories included in the global food balance sheet and about half of the protein. However, the current pace of grain yield increase through crop breeding is not sufficient to meet the ever-growing demand (Ray et al., 2012, 2013). The crop improvement community is thus under pressure to develop resource-use efficient, nutritious, and climate resilient cultivars.

Plants in natural environments are exposed to a wide range of external stresses during their life cycle, and to survive and reproduce they continuously integrate external and development cues to optimize their fitness, especially when resources are limited, at least within their genetic and adaptive limitations. Tradeoff refers to “*situations when one trait cannot increase without a decrease in another trait (or vice versa)*” (Garland, 2014). A variety of tradeoff exists, *inter alia*, source-sink, growth-defense, or yield-nutrition nexus. Plants under biotic stress divert more resources to expression of defense related traits at the expense of growth and reproduction (Herms and Matsson, 1992) and plasticity (Dwivedi et al., 2020) which typically implies tradeoffs-is the way plants adapt to their physical environment. A better understanding of mechanisms that drive this antagonism may provide opportunities to refine breeding strategies that ensure such tradeoffs favor crop productivity.

Plant breeders improve complex traits controlled by multiple genes with small effects such as yield and nutritional quality. They make selections based on multiple traits, often unfavorably interrelated, which may limit the progress in crop breeding (Falconer and Mackay, 1996). Selection of traits with shared antagonistic genetic influence is functionally constrained while correlations induced by linkage disequilibrium can be disrupted by recombination (Falconer and Mackay, 1996; Lynch and Walsh, 1998).

Most of the available literature on tradeoffs relates to growth-defense mediated by phytohormones (Huot et al., 2014; Morales and Munné-Bosch, 2016; Nguyen et al., 2019; Ning et al., 2017), phytohormones-based cross-talks and signals impacting multiple responses (Jang et al., 2020; Verma et al., 2016), rewired trait relationships due to domestication (Martin, 2021), and growth-defense linked cost mitigation (Karasov et al., 2017). This review article emphasizes the role of pleiotropy, linkage disequilibrium, phytohormones, and secondary metabolites in mediating tradeoffs; how genes and networks regulate tradeoffs; and options to mitigate tradeoffs in plant breeding for the development of resource-use efficient, productive, and nutritionally enhanced grain crops.

## Genetic tradeoff as influenced by pleiotropic effects and tight linkages

As noted from the perspective of agronomy and ecology by Sadras and Denison (2016), selection does not always lead to optimal solutions due to trade-offs–often unknown, variation across environments, plant development *per se*, and genetics (all of which may be correlated). According to them, both trade-offs and environmental variation do not allow single factor optimization, e.g., in plant architecture and physiology, or use of inputs. In their view, “optimality” should be defined by a composite defined function rather than by a single criterion. Genetic tradeoff in plant breeding refers, however, to a situation when improvement in one trait adversely impacts another trait and vice versa. Indeed, quantitative variation among traits often shows relationships, thus affecting plant breeding because improving one may affect the other. Furthermore, there are multi-functional proteins (known as moonlighting) performing various autonomous and not always related functions, e.g., a catalytic enzyme may be also participating in unrelated processes such as autophagy, protein transport, or DNA maintenance (Huberts and van der Klei, 2010). These moonlighting proteins are often performing unrelated functions because they do not partition such function in distinct protein domains. Likewise, there are other genes that are switched off without stress but show elevated expression under stress. For example, rice lacking *OsPQT3* (a homologue of *Arabidopsis PARAQUAT TOLERANCE 3*, which regulates negative oxidative stress responses, shows enhanced tolerance to both the non-selective herbicide paraquat and to salt stress (Alfatih et al., 2020). Grain yield of rice of *ospqt3* mutants increases in the field because they show more tillers and larger panicle size than the wild type (*OsPQT3*) plants.

The theory of quantitative genetics based on Fisher’s infinitesimal model (Fisher, 1919) considers many loci of small effects affecting phenotypic variation, which was demonstrated by association genetic research using genome wide analysis (Boyle et al., 2017 and references therein). Genetic correlations measure the strength of trait associations which may arise from either pleiotropy because of same genetic influence or linkage disequilibrium due to non-random association of alleles. Hence, both pleiotropy and tight linkages may account for observed tradeoffs among traits. It is worth indicating that Sadras and Denison (2016) consider pleiotropy as a tradeoff *per se* because the multiple effects of a given allele on different traits do not allow optimizing a genotype; i.e., a sort of “outbreeding depression” in the environment where the plant grows that may affect resource use (or ecological performance).

The association between breeding values (or the expected phenotypes of an individual’s offspring) among traits in a defined population evaluated in a target set of environments determines the genetic correlations (Lynch and Walsh, 1998). They are positive if both traits increase or negative when tradeoffs among them are noticed. The genetic correlation coefficients are used in plant breeding to predict responses to multi-trait selection, often through an index. These coefficients are estimated through covariance analysis among traits considering the resemblance among relatives or through the correlated response to selection if one of a pair of individuals is selected for one trait and the other individual for another trait. Although sampling errors may affect the estimates, some patterns may be observed, e.g., tradeoffs between fecundity and quality or edible yield vis-à-vis produce quality in some field crops. For example, rice, through crossbreeding, increased spikelet number per panicle in large-panicle plants, but this advantage did not improve grain yield because of poor filling in the inferior spikelet, thus suggesting a tradeoff between grain filling and spikelet number that relates to ethylene production and starch biosynthesis (Panigrahi et al., 2019).

## Physiological tradeoff limiting selection for stress tolerance, productivity, and nutritional quality in crop breeding

Tradeoffs in the expression of plant traits are an invariable consequence of their plasticity, thus helping maintain relative fitness under a wide range of unpredictable conditions (Sadras and Lawson, 2011; Sadras et al., 2009). The response allows the plant to adjust its needs to available resources, and in the case of annual species, maximize the probability of both reproduction and its fecundity (Lake et al., 2016). Since we typically consume the seed of staple crops, it is therefore to be expected that one of the most common trade-offs considered in breeding is that between ‘source’ and ‘reproductive sinks’, which ultimately determines seed yield for a given amount of total carbon assimilated (Aggarwal et al., 1990; Reynolds et al., 2005). In productivity terms, the ‘source:sink’ trade-off is represented by the harvest index (HI). Genetic gains in many crops including cereals–which provide up to 70% of total human calories–have been attributable to an improved HI, reflecting the introduction of semi-dwarfing genes of major effect, as well as those associated with reproductive timing that permit a more favorable HI depending mainly on latitude and sowing date (Fischer et al., 2014). In wheat, at least, further incremental gains in HI have accounted for most of the genetic gains in the last half of the 20^th^ century (Reynolds et al., 1999).

When it comes to further increasing HI, another pertinent trade-off is that between seed number and seed size (Labra et al., 2017; Sandras, 2007), traits directly associated with the probability of reproduction, and the likelihood of successful plant establishment, respectively. Much has been written over the decades about the apparent unbreakable trade-off between these two traits, since grain yield could be boosted significantly if this presumed genetic link were broken (Ferrante et al., 2017). Some promising results were found in wheat when a cross was made between two CIMMYT high yielding advanced spring wheat lines (‘Kauz’ and ‘Babax’) that contrast in grain size and number, resulting in a few segregants that outperformed both parents by a large margin through favorable expression of both yield components (Bustos et al., 2013; García et al., 2013). Perhaps ironically, the expression was not robust in the environment where parents were developed, perhaps due to a shorter growing seasons or shorter photoperiod (Griffiths et al., 2015). Now it seems that a breakthrough may have occurred using a transgenic approach to break the linkage (Calderini et al., 2021). Results showed that targeted overexpression of an α-expansin in early developing wheat seeds increased grain size without affecting grain number, thereby resulting in ∼12% higher average grain weight than the control under favorable field conditions and normal planting density. The breakthrough provides a potential model for overcoming the grain size *versus* grain number tradeoff that represents a persistent bottleneck to genetic yield gains across many crops, although of course overcoming such a limitation to yield can be expected to reveal new limits or trade-offs.

The issue of trade-offs among other plant organs–besides that between seed size and number–was firmly established by the Green Revolution in cereals, where reduction in stem length enabled crops to invest more resources in reproductive structures, increasing not only HI but yield potential as a result of an improved mechanical structure and therefore responsiveness to external inputs (Pingali, 2012). Interestingly, there is still direct evidence in wheat that stem growth in the internodes where their extension coincides with rapid spike growth competes with each other for resources (Rivera-Amado et al., 2019). These results identified decreased partitioning to stem internodes 2 and 3, as well as the rachis, but increased lemma partitioning, to be associated with increments in HI.

Another trade-off in cereals that is poorly understood is that between spike density/tiller number and the size of the reproductive organ (or spike) (Ferrante et al., 2017). As mentioned, high yielding wheat lines have been developed at both ends of and across that spectrum, where the former typically have small spikes and grains with a high spike density (e.g., ‘Kauz’ type) and the latter large spikes and grains with relatively low spike density (e.g., ‘Babax’ type); similar genetic variation is common across cereals. It remains unknown if there is an optimal spike density, albeit a function of environment and planting system, although in maize (where the largely uniculm growth habit permits well controlled experimentation with stand density), it was shown that modern hybrids perform better due to plant density tolerance (Duvick, 1992), indicating a clear genetic component. What is known and somewhat perplexing is the apparent overproduction of tillers in small grain cereals, akin to indeterminacy in other crops. Vigorous early tiller production is a way to maximize early light interception, potentially decrease the negative consequences of winter kill or other negative environmental factors, and store N that may be more easily taken up earlier in the season for later remobilization to growing tissue. Nonetheless, preliminary data in high yielding wheat lines have indicated a negative association between grain yield and tiller abortion from the heading stage onward, suggesting that overproduction of tillers can be negative to yield (Molero et al., 2019). More research is needed to understand the pros and cons of apparent over-fecundity in crop species in order to design better genetic targets (Sandras, 2007; Sadras and Denison, 2009).

Under severe abiotic stress, other trade-offs come into play. Being an outcrossing species, maize shows an ancestral tradeoff under stress, related to its reproductive biology. This is expressed as a delay in silking–while anthesis remains relatively unchanged–and is indicative of a reduced partitioning of assimilates to the developing cob (Bolaños and Edmeades, 1996). The evolutionary reasons presumably relate to ensuring that at least the pollen remains viable and within the broader gene pool under extreme stress. Clearly, the trait is undesirable agronomically and much effort has been invested in reducing expression of this ‘survival’ trait (Edmeades et al., 2017). One of the most obvious tradeoffs is under drought stress where a crop can invest scarce assimilates into roots to access subsoil water (Kirkegaard and Hunt, 2010) or restrict root access to more easily available water using efficient budgeting over the duration of their life cycle (Hall and Richards, 2013). This is often oversimplified as a trade-off between water uptake or water use efficiency, although, in practice, efficient use of water would represent the ideal balance between these (Blum, 2009). One study in wheat sister lines suggested a direct genetic trade-off between carbon investment in deep roots to access deep water, versus carbon storage in stems–as water soluble carbohydrates (WSC)(Lopes and Reynolds, 2010)–assimilates used later for filling grains. What is largely unknown is the potential trade-off between root growth and function with respect to the cost of supplying carbon to symbiotic microorganisms in the rhizosphere. It is known that soil environment and species affect composition and diversity of the soil microbiome (Latz et al., 2021), while root architecture, turnover, and exudates affect the composition and magnitude of microbiota (Sasse et al., 2018). Drought stressed plants change the composition of root exudates, increasing microbial activity (de Vries et al., 2019) which may aid in recovery, but much more research is needed to understand potential trade-offs under different cropping systems and environments.

The storage of WSCs in cereals represents another poorly understood trade-off, in fact. While WSC accumulation and remobilization have been found to buffer yield under stress (Blum, 1998; Rebetzke et al., 2008), they appear to represent waste of carbon under more favorable conditions, typically remaining in the stem at harvest. Presumably, their value as a buffer against unexpected adverse conditions during grain-filling is hardwired as a trait, while the large genetic variation seen among modern cereal cultivars suggests that the trait has yet to be fully explored in breeding, especially under high yield (Saint Pierre et al., 2010).

The root:shoot remains a poorly understood trade-off in most crops because roots are relatively poorly studied compared with above ground growth, for obvious reasons. Genetic variation in root:shoot has been established and linked to yield under heat and drought stress, in wheat for example, although distribution of root mass to where water is present is a more important variable (Gao and Lynch, 2016; Pinto and Reynolds, 2015). The effectiveness of root system architecture will ultimately determine the need for carbon investment (Ye et al., 2018). However, there is a clear need to understand these tradeoffs better and their interactions with the environment (Hutchings and John, 2004).

There is much debate about whether breeding for nutritional quality sacrifices yield potential. Simply based on probabilities, selecting for one trait is likely to be neutral at best for another discrete trait. However, there are clear cut cases where there is a metabolic cost of quality traits; for example, in the case of the energy requirement for synthesizing proteins or lipids stored in edible organs versus that of reserve carbohydrates. Despite that, genetic yield improvement does not have to come at the price of reduced quality (Guzmán et al., 2017). Nonetheless, yield is not the only consideration when it comes to quality related traits. In a study where quality was considered in terms of trade-off with nitrogen use efficiency (NUE), it was concluded that development of wheat genotypes that lack storage proteins–specifically those with no benefit to baking quality–could improve NUE, and as a result reduce the environmental footprint of its cultivation (Zörb et al., 2018). Under relatively extreme stress and where carbohydrates predominate in terms of seed composition–such as in cereal–while grains may achieve low filling rates in terms of starch, the germ is preferentially maintained for reasons of survival and this can actually benefit nutritional quality.

## Genetic and molecular basis of tradeoff to developing breeding populations and cultivars

### Plant growth-defense tradeoff as mediated by hormones

Plant hormones, also known as phytohormones, are signal molecules produced within plants that occur in extremely low concentrations. They are involved in plant growth, development, and stress tolerance. Abscisic acid (AA), auxins (AUX), brassinosteroids (BA), cytokinins (CK), ethylene (ET), gibberellins (GA), jasmonates (JA), salicylic (SA), and strigolactones (SLs) are major hormones. AUX, BR, CK, and GA are growth promoting phytohormones, while ABA, ET, JA, and SA are stress responsive phytohormones (Gray, 2004; Verma et al., 2016). Phytohormones play a critical role in mediating the tradeoff between growth and defense to optimize resources for sustained growth, stress tolerance, and productivity. Intensive crosstalk and fine-tuning between two groups of phytohormones optimizes plant growth, development, and defense (Hou et al., 2013; Li and Hou 2017; Yang et al., 2019). The exogenous application of JA, ET, SA, and CA or their functional analogs to plant impairs plant growth and development while boosting plants immunity to stresses (Dubois et al., 2018; Guo et al., 2018a; Huot et al., 2014). The tradeoff between plant growth and defense have been widely covered and published elsewhere, more so on biotic than abiotic stresses (Dolferus, 2014; Huot et al., 2014; Karasov et al., 2017; Riemann et al., 2015; Sah et al., 2016; Verma et al., 2016). Understanding the physiological basis of growth-defense tradeoffs in *Arabidopsis* and field crops during stresses may provide breeders the opportunity to simultaneously select for increased yield and adaptation to environmental stresses and raise food crops productivity.

***Arabidopsis:****Arabidopsis thaliana* is a model plant for unfolding the genetic and molecular basis of biological functions in plants (Somerville and Koornneef, 2002). Elevated defenses due to phytohormones are commonly associated with growth inhibition. A coexpression transcriptional network analysis in *Arabidopsis* treated with coronatine (COR), a toxin produced by the *Pseudomonas syringae*, which causes stomata to re-open in the night (Panchal et al., 2016), unfolded the core regulatory module in which the genes were rapidly activated and sustained upregulation after COR treatment to mitigate growth-defense tradeoffs. Several transcription factors (TFs) such as *RAP2.6L*, *MYB44*, *WRKY40*, and *WRKY18* were identified as instantly activated components associated with pests and diseases resistance. Jasmonic acid (JA) rapidly activates *RAV1* and *KAN1* to repress brassinosteroid responses genes, upregulate *KAN1*, the C2H2 TF families *ZF2*, *ZF3*, *ZAT6*, and *STZ/ZAT10* to repress the biosynthesis, transport, and signaling of auxin to arrest growth, providing a comprehensive snapshot of genes that respond to JA signals and may be harnessed to select for robust growth and defense simultaneously in breeding programs (Zhang et al., 2020b).

GA mediate plant growth and development, while crosstalk other phytohormones through DELLA proteins mediate the growth-defense tradeoffs (De Bruyne et al., 2014; Hou et al., 2013; Yang et al., 2012). DELLA proteins and EDS1, an essential resistance regulator, form a central module that mediates plant growth-defense tradeoffs in *Arabidopsis*. EDS1 in the event of pathogen infection rapidly promotes SA biosynthesis and resistance-related gene expression to prime defense response, while pathogen infection stabilizes DELLA proteins RGA and RGL3 to restrict growth in a partially EDS1-dependent manner, which facilitates plants to develop resistance to pathogens. The increasingly accumulated DELLAs interact with EDS1 to suppress SA overproduction and excessive pathogen response, thereby suggesting that plants via a DELLA-EDS1-mediated feedback regulatory loop maintain the subtle balance between growth and defense to avoid excessive growth or defense in response to pathogen attack (Li et al., 2019a).

Atypical E2F TF *DP-E2F-like1* (*DEL1*) and JASMONATE ZIM-DOMAIN (JAZ) proteins modulate growth-defense tradeoffs in *Arabidopsis*. Root-knot nematode (RKN) worldwide causes substantial losses to crop production. SA accumulation leads to the activation of plant defense responses. Nakagami et al. (2020) showed that *DEL1* represses excessive SA accumulation and root growth inhibition of host plants upon RKN infection in *Arabidopsis*. The *DEL1*-deficient mutant (*del1-1*) in contrast shows excessive SA accumulation and lignin in galls and is more resistant to RKN infection. This suggests that *DEL1* balance growth and defense responses to RKN infection by controlling SA accumulation and lignification.

JA-triggered depletion of JAZ proteins reduces growth and seed yield. JAZ-deficient mutants exhibit high levels of defense and strong growth inhibition. Major et al. (2020) uncoupled growth-defense tradeoffs in *jazD* mutant and identified 9 independent causal mutations in the red-light receptor phytochrome B (phyB). Unlike the ability of the phyB mutations to completely uncouple the mild growth-defense phenotypes in a *jazQ* mutant defective in *JAZ1*, *JAZ3*, *JAZ4*, *JAZ9*, and *JAZ10*, phyB null alleles only weakly alleviate the growth and reproductive defects in the *jazD* mutant. Furthermore, phyB-independent growth restriction of the *jazD* mutant is tightly correlated with upregulation of the Trp biosynthetic pathway but not with changes in central carbon metabolism, indicating that the mechanisms underlying JA-mediated growth-defense balance depend on the level of defense and association between growth inhibition at high levels of defense and dysregulation of Trp biosynthesis (Major et al., 2020).

Clearly, the snapshot of genes responding to JA signals in *Arabidopsis*, with similar function across plant species (Provart et al., 2016), are valuable resources for functional studies on the genetic modification of breeding population that exhibit robust growth and defense simultaneously.

***Grain crops:*** Alternative splicing isoforms in plant genes affect growth, development, and stress tolerance (Shang et al., 2017). *OsPDR1* encodes three splice isoforms: *OsPDR1.2, OsPDR1.3*, *OsPDR1.1*, with the former two containing a conserved glutamate residue in the “ENI-motif” of the first nucleotide-binding domain and the latter does not. *OsPDR1* transcripts in rice are developmentally controlled and differentially regulated by JAs and pathogen infection. The *OsPDR1.2*- and *OsPDR1.3*-overexpressing plants exhibit higher JAs content and stronger growth inhibition and disease resistance than *OsPDR1.1*-overexpressing plants. Thus, alternative splicing affects the function of *OsPDR1* in regulating growth-defense tradeoffs (Zhang et al., 2020a).

An *in vitro* experiment involving the rice cultivar ‘IR64’ and varying doses of exogenous JA application revealed that a dose of 5 μM reduces root length (RTL) by 60% and shoot length (SHL) by 40% (both trait measurements relative to the untreated control). A genome-wide association study involving a rice panel of 155 *indica* accessions screened at a dose of 5 μM JA for 10 days during germination unfolded substantial natural genetic variation and detected 28 significant associations for RTL, SHL, root weight (RTW), and total weight (TTW). Three common QTL for RTL, RTW, and SHL and candidate genes, including several JA-responsive transcription factors known to play a role in stress response were unfolded, which may be useful in breeding to optimize the growth-defense tradeoff in rice (To et al., 2019).

### Balancing tradeoff between multiple stresses, productivity, and quality

***Arabidopsis:*** Any abiotic or biotic stress alone or together decreases plant fitness. In this regard, research in the plant model system *Arabidopsis* shows that the locus *ACQOS*–controlling acquired osmotolerance–contributes to host plant resistance to bacteria in the absence of the osmotic stress, but in its presence *ACQOS* causes detrimental autoimmunity, thereby decreasing osmotolerance (Ariga et al., 2017). This research points out that *Arabidopsis* keeps functional and non-functional alleles in this locus due to the trade-off between abiotic and biotic stress adaptation. Such a finding suggests that some genes involved in host plant resistance or tolerance may be influenced by competing stresses.

The molecular mechanisms linking growth and plant immunity are not well understood. The hormone jasmonate–which regulates plant growth, development and defense–mediates some growth-defense tradeoffs. Campos et al. (2016) using *Arabidopsis* mutants in the analysis of epistatic interactions noticed that growth inhibition related to anti-insect resistance ensued through a conserved transcriptional network activated by jasmonate signaling rather than by diverting photo-assimilates from growth to defense.

***Grain crops:*** There seem to occur trade-offs between productivity and stress tolerance or host plant resistance. Nevertheless, their underlying causes are recently emerging. Heat affects negatively kernel number and accumulation of seed storage molecules (e.g., starch) in the endosperm during grain filling in maize, thus reducing grain yield of this crop under such a stress. Ribeiro et al. (2020) found that many kernels could develop under heat stress by modifying the high-temperature sensitive enzyme 6-phosphogluconate dehydrogenase acting in the central carbon metabolism, thus showing that this approach may facilitate adapting maize to global warming. Their research clearly provides insights regarding subcellular distribution of metabolic activities in the endosperm. For example, it seems that during kernel metabolism the amyloplast pentose phosphate pathway is a heat-sensitive step.

Zhang et al. (2020a) characterized *OsPDR1*, which is a JAs-inducible gene in rice encoding a member of the pleiotropic drug resistance subfamily of ABC transporters. They found that the overexpression of *OsPDR1* led to the constitutive activation of defense-related genes for resistance to bacterial blight, while a mutation decreased the host resistance to the pathogen. The overexpression and mutation of this gene decreased and increased early plant growth at seedling stage, respectively, but at the end decreasing grain yield. Their research also shows how *OsPDR1* isoforms are playing distinctly fine-tune growth and development, thus affecting productivity in rice.

Pleijel and Uddling (2011) analyzed trade-offs between grain yield vs. quality in wheat under stress brought by either carbon dioxide (CO_2_) or ozone (O_3_). They found that elevated CO_2_, but not O_3_, affected negatively grain protein yield even when stress effects were not noticed on grain yield. Their research also demonstrated that O_3_ affected grain mass–other quality trait–stronger than grain number, while the reverse was true for CO_2_. It was also noted that O_3_ negatively influenced harvest index, which was not affected by CO_2_. They concluded highlighting that the most important negative effect of CO_2_ was on grain protein accumulation independently of the grain yield effects.

### Secondary metabolites, a source of tradeoff between abiotic and biotic stress tolerance

Organic compounds such as toxins, secondary or natural products that are not directly involved in growth, development, or reproduction of a plant are known as secondary metabolites, which mediate plant–environment interactions (Erb and Kliebenstein, 2020). Secondary metabolites, which are multifunctional, include terpenes, phenolics, and nitrogen (N) and sulfur (S) containing compounds (Mazid et al., 2011). They defend plants against pathogens, pests, and abiotic stresses. Secondary metabolites are affected by the growth-differentiation balance; i.e., as indicated by Herms and Matsson (1992), the tradeoff between growth and defense arises due to physiological constraints between secondary metabolism and structural reinforcement in dividing and enlarging cells. Plants should accelerate their growth and maintain the necessary defenses under stress.

Species, genotype, physiology, development stage and the environment affect both concentration and type of plant secondary metabolites (Isah, 2019). The fitness-costly responses of crops to conflicting stresses (that often result in a yield penalty) leads to physiological tradeoffs due to phylogenetic constraints as recently shown by Montesinos-Navarro et al. (2020). Using multivariate analysis, they found investment tradeoffs among species’ responses to abiotic and biotic stresses. For example, species responding to an abiotic stress (proline and abscisic acid contents) trade off against their investment to face a biotic stress (jasmonic and salycic acids). They also noticed an evolutionary conserved metabolism among closely related species, which suggests including plant evolutionary history when doing physiological research under stress, thus gaining insights in their responses to various stresses occurring in the agroecosystems.

Plants may be affected by various stresses simultaneously, which calls for further research because it is necessary to understand their interactions in a changing climate. For example, *A. thaliana* prioritizes differently its distinct-age leaf responses to keep growth and reproduction under coincident abiotic and biotic stresses (Berens et al., 2019). It seems that in *A. thaliana* a genetic mechanism balances any tradeoffs arising from the conflicting stress and the interactions among abiotic stress tolerance, host plant resistance, and the leaf microbiota.

Phytohormones regulating organismal processes and metabolism appear to be involved on alleviating stress in crops, e.g., salicylic acid for abiotic stress tolerance (Khan et al., 2015). The regulation of plant secondary metabolism following interactions between heat shock and elevated CO_2_ was investigated in willow (*Salix* spp.) using an untargeted metabolomic fingerprinting approach (Austen et al., 2019). This research demonstrated that isoprene biosynthesis continues under both high temperature and elevated CO_2_ with the former having the greater effect.

Omics data analysis along with functional genomics are providing insights regarding the junction of signaling pathways for joint abiotic and biotic stress adaptation across various cellular compartments and also when considering the whole plant (Kissoudis et al., 2014). For example, elicitors tacking drought triggered the salicylic acid pathway but induced susceptibility to the chewing insect *Ascia monuste* in broccoli and *Arabidopsis* (Venegas-Molina et al., 2020). Dissecting stress tolerance along with host plant resistance may therefore provide new insights into both stress cross-regulation and target genes for further breeding under simultaneous stresses.

### QTL and candidate genes regulating trait tradeoff in *Arabidopsis*

#### Optimizing source‒sink‒flow transport

In plants system, the ‘source’ refers to any tissue (leaves and other green tissues) that produces photoassimilates, while ‘sink’ relates to any tissue (roots, tubers, fruits, seeds) that is the net importer of photosynthetic products (C, N). ‘Flow’ refers to the transport system connecting ‘source’ and ‘sink’ tissues. Are plant growth, development, and yield source- or sink-limited? Does there exist any tradeoff between the two processes? Genetic and molecular networks controlling the resource distribution and partitioning to competing organs are very complex, but it appears to be highly fine-tuned to respond to distinct growing environments, thereby enabling plasticity.

Plant uses light energy to convert CO_2_ into carbohydrates. Starch accumulates in the light and is degraded at night to provide a sustained supply of C for plant growth. Starch turnover and C allocation in *Arabidopsis* occupy a central role in the network that coordinates metabolism with growth. Sulpice et al. (2009) noted coordinated changes in transcripts of more than 70 C-regulated genes, with two genes, *myo-inositol-1-phosphate synthase 1* (*IPS1*) and a Kelch-domain protein, as candidate with potential to increase biomass production. Furthermore, association analysis revealed polymorphisms in these genes to relate with biomass and show opposite allelic effects on metabolites, which may be used directly or through the isolation of homologs to modulate biomass production in crops.

Sucrose is the major carbohydrate produced during photosynthesis and transported to sink tissues via the phloem cells of the plant’s vascular system. This long-distance transport of sugar is mediated by pressure-driven mass flow of a large osmotic gradient generated by a proton-sucrose symporter (Bush, 2020). *SUCROSE TRANSPORTER 2* (*SUC2*) or its homologs, whose activity is controlled via its protein turnover rate and phosphorylation state, regulate the rate of carbon export from source leaves into the phloem vascular tissues in most crops including the model plant *Arabidopsis*. *UBIQUITIN-CONJUGATING ENZYME 34* (*UBC34*) trigger turnover of *SUC2* in a light-dependent manner. *ubc34* mutants showed increased phloem loading and increased biomass and yield, while mutants of another SUC2-interaction partner, *WALL-ASSOCIATED KINASE LIKE 8* (*WAKL8*) had decreased phloem loading and growth, thereby suggesting that both proteins are required for the up-regulation of phloem loading in response to increased light intensity, and a promising target for enhancing source strength (Xu et al., 2020).

#### Carbon (C):nitrogen (N) partitioning

The term ‘orphan’ gene refers to a subset of protein-coding genes lacking recognizable homologs in other organisms (Arendsee et al., 2014). C and N play an important role in the synthesis of plant proteins, carbohydrates, and lipids. The severe negative tradeoff between seed yield and protein often poses a major challenge to effect simultaneous improvement in crop breeding (Simmonds, 1995). *QQS* is an *Arabidopsis*-specific orphan gene that affects C partitioning to both starch and protein (Li et al., 2009).

#### Growth versus defense

Plants being sessile in nature often experience multiple stresses, both abiotic and biotic, and therefore are required continuously to prioritize either growth or defense responses to survival and reproduction. The literature suggests that activation of defense responses occur at the expense of growth, which is termed as growth-defense tradeoff (Huot et al., 2014). This tradeoff is attributed to competing demand of energy allocation to growth and adaptation responses. Understanding the genetic and molecular basis of this tradeoff may facilitate ameliorating this negative tradeoff to optimize stress tolerance and productivity in new cultivars.

The *Arabidopsis* growth-related transcription factor HBI1 regulates apoplastic ROS homeostasis by differentially controlling the expression of NADPH oxidases (NOXs) and peroxidases (POXs). HBI1 target *RbohA* and *RbohC* genes. HBI1-induced *RbohC* promotes leaf cell expansion, while HBI1-repressed *RbohA* negatively regulates growth but promote disease resistance. Hence, the incompatibility between growth and defense is linked to the differential regulation of apoplastic ROS homeostasis during both processes (Neuser et al., 2019).

Nitrous oxide (NO), a small gaseous molecule, is key regulator of diverse biological functions in plants (Besson-Bard et al., 2009; Durner and Klessing, 1999; Gayatri et al., 2013; Shi et al., 2014). ABA-metabolism-related genes show differential expression in response to NO donor S-nitroso-L-cysteine (CySNO) (Imran et al., 2018). Khan et al. (2019) identified CySNO-induced ABA-related genes and noted loss of function mutant *atao3* differentially regulate oxidative and nitrosative stresses. The *atao3* plants showed resistance reaction to *Pseudomonas syringae*, due to gradual increase in *PR1* gene expression. The *agsnor1-3* and *atsid2* mutants were susceptible because of reduced *PRI* transcript accumulation. The *atao3* and *atnced3* ABA-deficient mutants showed early wilting and eventually plant death as their stomata remained open even at 7 days after drought stress. Research suggests several TFs including OsbHLH034 regulate JA-mediated resistance response against bacterial blight in rice. In this regard, *OsbHLH034* overexpressing transgenic rice plants showed enhanced resistance to bacterial blight but were overly sensitive to salt stress (Onohata and Gomi, 2020).

#### Translating insights from Arabidopsis to grain crops

*Arabidopsis thaliana* has been most extensively studied model plant for unfolding plant biology functions and responses to environment. Many basic discoveries made using this plant have empowered the research community to unfold similar functions in higher plants. No single plant species fully embodies the features of all other species. Approximately two-thirds of *Arabidopsis* gene families (9503), for example, share with poplar (*Populus trichocarpa*) (13,144), sorghum (*Sorghum bicolor*) (16,378), and rice (15,148) (Woodward and Bartel, 2018). The receptors and signaling pathways of almost all plant hormones, however, have been elucidated in *Arabidopsis* that often function similarly across plant species (Provart et al., 2016). A few functionally characterized genes regulating tradeoff have recently (2019–2021) been unlocked in *Arabidopsis* (Table 1), whose functions (or discovering homologs) in higher plants are yet to unfold. Not all the knowledge gained from *Arabidopsis*, however, can be transferred in applied breeding of grain crops.

**Table 1:** Genes regulating source *versus* sink, carbon (C):nitrogen (N) partitioning, and growth versus defense tradeoffs in *Arabidopsis*

Gene	Description	Reference
**Source versus sink**		

*UBC34, WAKL8*	Increased C loading in the phloem by *ubc34* result in greater biomass and yield, while decreased C loading by *WAKL2* mutants reduces growth	Xu et al. (2020)

**C:N partitioning**		

*QQS*	Regulate C partitioning to both starch and protein	Li et al. (2009)

**Growth versus defense**		

*AITR1-6*	Enhanced drought and salt resistance in knockout *aitr256* triple, quadruple *aitr1256*, or sextuple *aitr123456* mutants with no adverse impact on plant growth and development	Chen et al. (2021)
*RbohC*, *RbohA*	HBI1 TF-induced *RbohC* promotes leaf cell expansion, while HBI1-repressed *RbohA* negatively regulates growth but promotes disease resistance	Neuser et al. (2019)
CySNO-induced ABA-related genes	Loss of function mutant *atao3* differentially regulate oxidative and nitrosative stresses; *atao3* plants resistant to *Pseudomonas syringe* due to gradual increase in *PR1* expression, while *agsnor1-3* and *atsid2* mutants susceptible due to reduced *PR1* transcript expression	Khan et al. (2019)

Species within grasses with plant biology and genome size close to commercially grown cereal crops should be explored as model for cereals. The genus *Brachypodium* is an interesting model system that has advanced our knowledge of the biology of grasses. *Brachypodium distachyon*, a C_3_ plants distributed worldwide, fits very well as a model plant for unfolding cereal biology because of its small genome (∼272 Mb), short life cycle, small stature, amenability to genetic transformation, and suitability for laboratory and field experimentation (Scholthof et al., 2018).

Maize, rice, and wheat together provide half of the food to humankind (Alexandrator and Bruinsma, 2012). Unlocking the biology of these species, however, proved challenging due to their large size, long life cycle, and large genome. Advances in gene editing, speed breeding, and genome assembly techniques besides improved transformation protocols, structured natural populations, sequenced mutant populations, and genome sequences are enabling researchers overcome challenges associated with working on such crops. Thus, there are attractive experimental systems of their own with which to make discoveries that are directly applicable to increasing crop production (Adamski et al., 2020; Borrill, 2019).

### QTL and candidate genes regulating trait tradeoff in grain crops

#### Optimizing source‒sink‒flow transport

Cytokinins and gibberellins (GAs) play antagonistic roles in regulating reproductive meristem activity in rice; i.e., GAs negatively affect it while an increased cytokinin activity leads to high seed number. *Grain number per panicle1* (*GNP1*), which encodes gibberellin biosynthesis gene *GA20ox1*, affects seed per panicle in rice (Wu et al., 2016). To unfold *GNP1* effect on sink, source, and flow in regulating grain yield in rice, Zhai et al. (2020) compared ‘Lemont’, a *japonica* cultivar, with its near isogenic line (NIL-GNP1^TQ^) in ‘Lemont’ background, containing an allele at *GNP1* locus from a high-yielding *indica* cultivar ‘Teqing’. NIL-GNP1^TQ^ produced on average ∼33% more grains per panicle and 7% greater yield than ‘Lemont’ by compensating for reduced seed setting rate, panicle number and single-grain weight. Likewise, more filled grains panicle^−1^ and greater vascular system–contributing to photoassimilates transport to spikelets–resulted in increased grain yield in NIL-GNP1^TQ^. The superior spikelets (SS) and inferior spikelets (IS) of NIL-GNP1^TQ^ in comparison to ‘Lemont’ showed significant differences in grain weight. The reduced grain weight of SS was due to decrease in grain size while both grain size and poor grain filling contributed to reduction in IS grain weight. The reduced activities of key enzymes associated with carbon metabolism could account for the poor grain filling in IS which resulted in more unfilled grains or small grain bulk density in NIL-GNP1^TQ^. Significantly lower carbohydrate accumulation in culms and leaf sheath before heading in comparison to ‘Lemont’ contributed to low seed setting rate and grain weight of IS in NIL-GNP1^TQ^. However, significantly increased grain number panicle^−1^ from introgression of *GNP1*^*TQ*^ into ‘Lemont’ did not result in significant improvement in grain yield of NIL-GNP1^TO^ primarily due to significant low sink activities in IS and insufficient source supply, not sufficient to meet the increased sink capacity demand (Zhai et al., 2020).

Seed yield is largely dependent on variation and relationship between source and sink. Using phenotyping (two environments) data on source (flag leaf length, flag leaf width, flag leaf area), sink (spikelets panicle^−1^, 1000-grain weight), source-sink relationship (spikelets to flag leaf area ratio) and yield related traits (grains panicle^−1^, panicles plant^−1^, grain yield plant^−1^, biomass yield plant^−1^, harvest index) and genotyping (469,377 SNPs) data on 272 *indica* rice accessions, Wang et al. (2020) reported 70 QTL in four chromosomal regions influencing 11 traits. Five QTL (*qHI6*, *qTGW7*, *qFLA8*, *qFGN1.2*, *qFLL1*), detected consistently in four chromosomal regions in both environments, simultaneously affected source, sink, source-sink relationship, and seed yield traits. Twenty-four candidate genes, including *NOG1*, *qH16*, *qTWG7*, and *qFLA8*, were co-located within the vicinity of these four consistent QTL regions, making these regions the ideal choice to manipulate source-sink-yield relationship for developing high-yielding rice cultivars by genomic-aided breeding.

#### Carbon (C):nitrogen (N) partitioning

The seeds from T_4_ generation transgenic soybean plants containing *QQS* and grown under growth chamber, greenhouse, and field conditions showed up to 18% increased protein and up to 13% less oil, with no adverse impact on plant growth, seed yield per plant, seed morphology or seed weight (Li and Wurtele, 2015). The transgenic maize, rice, and soybean containing *QQS* showed increased protein. *QQS* effect on increase in soybean protein was independent of the genetic background and original protein content of the cultivar (Li et al., 2015). *QQS* may be therefore deployed in breeding programs to alter seed composition in agriculturally diverse field crops without adversely impacting seed yield.

#### Seed yield versus duration

Crop breeders often notice negative tradeoff between growth cycle duration and productivity. Early maturing cultivars usually produce less than those with longer duration. Such cultivars suffer yield penalty from shortened vegetative growth periods. Thus, combining ‘high-yield’ and ‘early maturity’ in new cultivars is a significant breeding challenge. *Early flowering-completely dominant* (*Ef-cd*) is a major maturity duration regulatory gene in rice. *Ef-cd* encodes a long noncoding RNA (IncRNA) that is transcribed from the antisense strand of *OsSOC1*, which encodes a flowering activator in rice. *Ef-cd* positively regulates the expression of *OsSOC1* by affecting the chromatin modifications around *OsSOC1* locus, thus leading to the early maturity phenotype (Yu and Qian, 2019). Natural variation in *Ef-cd* locus were recently discovered and deployed to overcome this negative tradeoff. In a field test, early maturing *Ef-cd* NILs with their wild types as well as of the derivative early maturing hybrids with their wild type hybrids evaluated across latitudes shortens maturity duration by 7–20 days without a concomitant yield penalty (Fang et al., 2019). Thus, natural variation in *Ef-cd* locus could be exploited to genetically balance early maturity and productivity in rice.

#### Seed number and weight

Seed weight and number, the primary components of yield in field crops, are often negatively correlated. The seed number relative to seed weight is more genetically variable and highly plastic in response to environmental variation (Sandras, 2007). Understanding the genetic-physiological-molecular basis of this tradeoff may therefore facilitate to realize new levels of yield targets through cross-breeding.

Golan et al. (2019) fine-mapped a QTL associated with the *GNI-A1* gene that regulates floret fertility and seed weight. *GNI-A1* allele increases weight by suppressing distal florets and altering assimilate distribution of proximal seeds in basal and central spikelets. A rare polymorphism in F_2_ population from a cross involving wild emmer accessions was associated with seed weight, independent of seed number. ‘Zavitan’, which carries the rare allele, may be deployed for making simultaneous selection for increased seed weight and number to enhance seed yield in wheat. Thus, 1B allele from bread wheat cultivar ‘Weebill’ and *GNI-A1* allele from wild emmer accession ‘Zavitan’ are attractive targets to minimize seed weight and number tradeoff in wheat breeding. Stress constrains cell expansion in plants. The targeted overexpression of α-expansin–a protein with significant role in plant growth and development by relieving stress in the cell wall–in early seed development yielded 12% higher average seed weight and 11% increase in seed yield in a field experiment compared to the wild type (WT), thus indicating there was no tradeoff between the two yielding attributing traits in wheat and possibly in other grain crops (Calderini et al., 2021).

*GSN1* is a negative regulator of seed weight but a positive regulator of seed number per panicle, linked through a conserved MAPK cascade (OsMKKK10-OsMKK4-OsMPK6), in rice. Reduced expression of *GSN1* resulted in heavier but fewer seeds per panicle, whereas its overexpression increased seed number per panicle but with reduced seed weight, suggesting that the rice OsMKKK10-OsMKK4-OsMPK6 cascade inactivated by *GSN1* confers a distinct role in specifying the tradeoff between seed weight and number in rice (Guo et al., 2018b). Spikelets per panicle (SPP) largely influence seeds per panicle. A QTL locus *SGPD7*, like *FZP* (*FRIZZY PANICLE*) which represses the axillary meristems, confers dense panicle but with small seeds. The CNV-18bp duplication in ‘Chuan 7’ decreased *FZP* expression, prolonged panicle branching period and increased seed yield by coordinating the tradeoff between SPP and 1000-seed weight (Bai et al., 2017). Huo et al. (2019) showed that *NUMBER OF GRAINS 1* (*NOG1*) increases seed yield in rice by increasing seeds panicle^−1^ without any impact on panicles plant^−1^ or seed weight. *NOG1* introgression increases seed yield by ∼26% in the *NOG1*-deficient rice cultivar ‘Zhonghua 17’, whereas its overexpression in NOG1-containing ‘Teqing’ further enhances seed yield by ∼19%.

#### Seed yield vs quality

Variation in proteins, carbohydrates, and lipids determine the seed quality. High protein negatively impacts seed yield. The inverse relationship between oil and protein limits the breeder’s ability to effect simultaneous improvement. Both genetic (type of gene action, pleiotropy and linkage drag) and environmental factors including levels of N application influence the antagonistic effect between seed protein and yield, for example, there is no inverse relation between grain yield and protein when the high protein ‘Egan’ wheat cultivar was grown at plots with N above 100 kg ha^−1^, while there was an inverse relationship in ‘Egan’ in an N-limiting environment (Torrion et al., 2019). How may plant breeding address this negative tradeoff? The use of the index grain protein deviation (GDP) is one such approach to break up the negative correlation. This index enables the identification of wheat genotypes that show a higher-than-expected protein content at any given yield level (Monaghan et al., 2001; Oury and Godin, 2007). Furthermore, the discovery of additive and dominance effect QTL with strong evidence of pleiotropy having antagonistic effects led to suggest that genomic selection based on the index GDP may be a promising method to alleviate the inverse relationship between these traits in wheat breeding (Thorwarth et al., 2019).

Developing cultivars with greater oil and protein contents is a significant breeding challenge. In a recent genome wide association study in soybean involving over 600 accessions (MGs I-IV) and 34,104 SNPs, Lee et al. (2019) noted three and five genomic regions that were associated with seed protein and oil contents, respectively. A QTL on chromosome 5 increased oil with no effect on protein content, while another QTL on chromosome 10 increased protein content with little effect on oil content. These QTL after validation may be deployed in soybean breeding to ameliorate negative tradeoff between these two traits.

Limited supply of carbon during seed maturation results in tradeoffs among seed quality traits. The allocation of carbon for storage reserves changes during late stage of seed development, for example, protein and lipid levels decline while concentrations of indigestible raffinose family of oligosaccharides (RFOs) increase, which result in a decreased crop value. Using fast neutron mutagenized soybean populations with deletion in central carbon metabolic genes, Kambhampati et al. (2020) selected two lines, FN300012 and FN301952, with concurrent increases in oil and protein, by a combined 10%. Temporal changes in biomass composition revealed a delayed carbon allocation to RFO synthesis in the mutant lines compared to WT, thus showing to be a useful genetic resource to deploy in soybean breeding when simultaneously selecting for increased oil and protein contents.

#### Growth versus defense

Drought and heat stress, which often occur simultaneously, cause substantial yield losses to cereal production. Plants respond to combined stress in a unique way that cannot be predicted based on individual stress performance. However, simultaneous selection for multiple stresses and combining it with productivity is challenging because of possible growth-defense tradeoff. A GWAS study in wheat with 277 diverse accessions phenotyped across 30 environments (nonstress, drought-stressed, heat-stressed, and drought-heat-stressed) and genotyped with 395,681 SNPs, Li et al. (2019b) noted 295 loci associated with agronomic and eurytopic loci for multiple abiotic stress tolerance, many with consistent effect across different treatments. Six of these loci were simultaneously associated with agronomic traits and abiotic stress adaptation. The increased frequency of superior alleles controlling yield-related traits in the four loci in the last few decades diminished alleles controlling abiotic stress tolerance in the same loci. *TraesCS6A02G124100* and *TraesCS6D02G114400* control both seed yield and multiple stress tolerance and may facilitate unraveling the underlying mechanism of stress tolerance-productivity tradeoff in wheat (Li et al., 2019b).

*OsALDH2B1* acts as a master regulator of the growth-defense tradeoff in rice. *OsALDH2B1* expression in nonreproductive organs maintains a balance of the biological processes, while alteration (loss of function) of its normal function causes an imbalance among defense, growth, and reproduction (Ke et al., 2020). The rice immune sensor *XA21* acts as a mediator for stress protection and plant growth under water-limiting conditions. *XA21* expression increases deposition of lignin and cellulose in the xylem vessels that help plants survive drought stress (Shamsunnaher et al., 2020). Likewise, *OsPQT3* knockout mutants (*ospqt3*) in rice displayed enhanced resistance to stresses with elevated expression of *OsGPX1*, *OsAPX1*, and *OsSOD1*, and showed greater yield compared to WT under salt stress in greenhouse and field environments (Alfatih et al., 2020).

Overall, genes (or their orthologs) mitigating tradeoffs (Table 2), for example, source vs. sink, C:N partitioning, yield vs. duration, growth vs. defense, grain number vs. weight, or yield vs. quality may be deployed in ameliorating tradeoffs in crop breeding in cereals.

**Table 2:** Genes regulating growth *versus* defense, source *versus* sink, carbon (C):nitrogen (N) partitioning, earliness *versus* yield, and seed number*versus* weight tradeoffs in grain crops

Gene	Description	Reference
**Growth *versus* defense**		

*OsPQT3*	Rice knockout mutants (*ospqt3*) relative to wild type (WT) displayed greater resistance and higher yield in abiotic stress environments and the gene switched off when stress is relieved	Alfatih et al. (2020)
*OsALDH2B1*	A master regulator of the plant growth and abiotic stress adaptation in rice	Ke et al. (2020)
*OsbHLH034*	Transgenic rice overexpressing *OsbHLH034* showed enhanced resistance to bacterial blight but ultra-susceptible to salt stress	Onohata and Gomi (2020)
*XA21*	Acts as a mediator for stress protection and plant growth under water-limiting conditions in rice	Shamsunnaher et al. (2020)
*TraesCS6A02G124100*; *TraesCS6D02G114400*	Regulates plant growth, development, productivity, and multiple stress adaptation in wheat	Li et al. (2019b)
*IPA1*	Defends rice plants against fungal infection when needed but reallocates resources within days back to growth, sustaining both pathogen defense and crop yields	Wang et al. (2018)

**Source *versus* sink**		

*GNP1*	Regulates seeds per panicle and balances sink‒source‒flow to minimize negative tradeoffs in rice	Zhai et al. (2020)

**C:N partitioning**		

*QQS*	Transgenic soybean containing *QQS* enhanced seed protein by 18% and reduced oil by 13% with no adverse impact on plant growth, seed yield, seed morphology or seed weight	Li and Wurtele (2015)
*QQS*	Enhanced protein in transgenic maize, rice, and soybean containing *QQS* independent of genetic background and original protein content	Li et al. (2015)

**Earliness *versus* yield**		

*Ef-cd*	A major gene regulating maturity duration in rice (Yu and Qian, 2019); deployment of allelic variation in *Ef-cd* locus resulted in 7–20 days early maturity in NILs and derived early maturing hybrids across latitudes without penalty on yield	Fang et al. (2019)

**Seed number *versus* weight**		

*GNI-A1*	Regulates floret fertility and seed weight; a rare allele from ‘Zavitan’ (wild emmer) and 1B allele from ‘Weebill’ (bread wheat) associated with seed weight, independent of seed number, attractive targets to minimize seed weight and number tradeoff in wheat	Golan et al. (2019)
*NOG1*	Enhances seed yield in rice by increasing seeds panical^−1^ with no adverse impact on panicles plant^−1^ or seed weight	Huo et al. (2019)
*GSN1*	Negative regulator of seed weight but a positive regulator of seeds panicle^−1^; reduced *GSN1* expression resulted in heavier but fewer seeds panicle^−1^, whereas its overexpression increased seeds panicle^−1^ but reduced seed weight	Guo et al. (2018b)
*SGPD7*	An 18bp CNV duplication in wheat cultivar ‘Chuan 7’ increases panicle branching period and seed yield by coordinating the tradeoff between spikelets panicle^−1^ and seed weight	Bai et al. (2017)

## Biotechnology-led approaches to minimize tradeoff in plant breeding

### Wild and weedy relatives as resource to enhance resource use efficiency

Finding the genes for target trait(s) in crop gene pools is a first step for identifying plant germplasm sources that may improve resource use efficiency. Their genetic basis may be elucidated by a genome wide study and further use this knowledge for a genomic-led breeding approach. Most of available literature in the use of crop wild (and weedy) relatives refers, however, to host plant resistance, abiotic stress tolerance, produce quality and yield potential (Dwivedi et al., 2017), thus lacking thorough information on their potential for enhancing input use efficiency. Hence, the search for resource use efficiency traits may consider evaluating other genetic resources such as feral types, which are defined as crop-derived plants found outside agricultural fields where they survive and reproduce without management (Gressel, 2005). There are well known feral congeners or weeds of crops such as alfalfa (*Medicago sativa*), cassava (*Manihot esculenta*), cotton (*Gossypium arboreum*), creeping bentgrass (*Agrostis stolonifera*), oat (*Avena sativa*), oilseed rape (*Brassica napus*), radish (*Raphanus sativus*), rice, sorghum (*Sorghum bicolor*), sugar beet (*Beta vulgaris*) or wheat, among others. Feral plants do not benefit from irrigation or pest management or show poor agronomic fitness. Their seedlings also need minimal plant competition to establish and survive outside cultivation. Further introgression from feral types to crops may provide new sources of variation for use while breeding for resource use efficiency through increasing their resilience in low-input agroecosystems. In wheat pre-breeding a number of traits have been brought from wild species or landraces into elite backgrounds, including increased RUE under heat stress and yield potential conditions (Molero et al., 2019; Reynolds et al., 2017) and deeper roots under drought (Reynolds et al., 2007).

### Predicting breeding values and parental selection

Plant breeders often make too many crosses to generate breeding populations to select the best performing offspring in cultivar development. However, in practice less than 1% crosses yield superior lines. What is the probability that cross ‘x’ will yield superior offspring? The value of a cross depends on the performance of its best offspring than on its mean progeny performance. What is the best way to predict which among the possible crosses are likely to yield superior lines? Yield is a function of cumulative and interactive effects of multiple component functional traits. How loosely or tightly interrelated (positive or negative) or independent are the traits? What combination, elite × elite or elite × unadapted germplasm will generate offspring with superior mean? Also, what is the probability that cross ‘x’ will yield transgressive segregants?

Population mean and genetic variance may be used to discriminate among potential crosses based on expected mean of the selected progeny by using either ‘usefulness criterion’ or ‘superior progeny means’ (Zhong and Jannink, 2007). Usefulness of the cross (m) depends on the following relationships

$${\text{U}}_{\text{m}}=\mu_{\text{m}}+\Delta {\text{G}}_{\text{m}}=\mu_{\text{m}}+ί\sigma_{\text{G}\left(\text{m}\right)}{\text{h}}_{\text{m}}$$

where μ_m_ is the population mean of the homozygous lines derived from cross ‘m’, σ^2^_G(m)_ is the genetic variance among these lines, h_m_ is the square root of the heritability, and ί is the standardized selection intensity, while superior progeny mean is defined as:

$${\text{S}}_{\text{m}}=\mu_{\text{m}}+ί\sigma_{\text{G}\left(\text{m}\right)}$$

where S_m_ equates to U_m_ with a heritability of 1. Whether parental selection could be used to predict μ, σ_G_, superior progeny means (μ_sp_), and genetic correlations in breeding populations? Mohammadi et al. (2015) uses simulation and marker and phenotyping data on yield and deoxynivalenol (DON) on training population in barley to calculate genomic estimated breeding values (GEBVs) of recombinant inbred lines (RILs), which they named ‘PopVar’ and available as an R package, to predict μ_m_, σ^2^_G_, μ_sp_, and the correlated responses of multiple traits for biparental population. μ explained 82 and 88% of variation in μ_sp_ for yield and DON, respectively, and by including σ_G_ to the regression model, R^2^ values increased to 99.5 and 99.6%.

Quantitative traits are often genetically correlated which may retard progress from selection. Parental combinations that deliver more favorable genetic correlation (r_G_) may permit simultaneous selection for multiple traits. Using simulations to assess the genome-wide prediction of r_G_ and long-term response to selection when identifying crosses based on such predictions, Neyhart et al. (2019) noted moderate accuracy to predict r_G_. Heritability, population size, and cause of genetic correlation (pleiotropy or linkage disequilibrium) influenced predictions. The r_G_ prediction accuracy on real data of 26 barley breeding population ranged from −0.12 to 0.42, depending on trait complexity. Choosing crosses based on predicted r_G_ increased multi-trait genetic gain by 11–27% compared to selection on the predicted cross mean. More importantly, such cross selection in case of negative association mitigated or prevented unfavorable response in the trait under indirect selection. Hence, prioritizing crosses based on predicted r_G_ may be an effective approach to improving unfavorably correlated traits to enhance breeding efficiency.

Mackay et al. (2021) consider that plant breeding “works” due to both transgressive segregation and heterosis as a result of the dispersion of favorable alleles. This viewpoint therefore calls for a quantitative breeding approach such as using genomic estimated breeding values (GEBV) for selection (Desta and Ortiz, 2014), which are being used in both inbred line development and for producing hybrids. Nonetheless, as noted by Abbai et al. (2020), the GEBV for selection should fit into an agroecological genetics framework because crop improvement should rely on community performance rather than on individual plant fitness.

### Multi-trait genomic selection to minimize tradeoff in breeding

Plant breeders when doing selection consider multiple characteristics that may be related by genetic correlations. If a pair of characteristics are positively correlated then any selection type improves both, but negative correlations lead to an unfavorable response when selecting on another characteristic. It was not surprising that in the mid-1930s, Smith (1936) proposed a discriminant function for plant selection. In his view, “the value of a plant may be expressed as a linear function of its characters”, which according to him “will be the best available guide to the genetic value of each line”. A few years later the genetic basis for this selection index was described by Hazel (1943). Henderson (1963) further elaborated on the genetic advance when using a selection index in breeding. A linear selection index (LSI) based on phenotypic values is obtained as:

$$I_{j}=\;b_{1}{\overset{ˇ}{P}}_{1J}+\;b_{2}{\overset{ˇ}{P}}_{2J}+\ldots +\;b_{m}{\overset{ˇ}{P}}_{mJ}=\sum_{i}^{m}b_{i}\overset{ˇ}{P_{iJ}}$$

where b_i_and ${\overset{ˇ}{P}}_{J}$ are the weight of the phenotypic value and the mean phenotypic value of family j, respectively. Recently, Céron-Rojas and Crossa (2018) reexamined the theory behind the LSI and its practice in plant breeding. According to them, the LSI “allows extra merits in one trait to offset slight defects in another”, thus saving for further crossbreeding individuals with very high merit in one trait even if they are slightly inferior for other traits. The use of GEBV for selecting multiple traits follows the same biometric approach as per the LSI. Table 3 provides some examples of the recent use of multi-trait genomic prediction models in plant breeding. The available results suggest that often both prediction accuracy and selection gain increase by adding correlated trait measurements in genomic prediction models, although computational requirements may remain a shortcoming limiting its practical application. Nevertheless, Runcie and Cheng (2019) indicated that a naive cross-validation strategy used for addressing multi-trait prediction may be biased and could lead to sub-optimal choices between single and multi-trait models if secondary traits measured in the testing individuals are included for predicting target traits. Hence, an appropriate cross-validation strategy must be pursued to determine reliably if combining information from multiple traits may be useful.

**Table 3:** Examples on the use of multi-trait genomic prediction models in plant breeding

Crop	Description	Reference
Barley	Selecting crosses according to predicted genetic correlation may be effective for improving negatively correlated traits	Neyhart et al. (2019)
Maize	Multi-trait models were always better than their univariate counterparts in a single testing environment. They also improved predicting the performance of hybrids not yet evaluated in any environment	de Oliveira et al. (2020)
Soybean	If grain yield weighs the selection for superior genotypes, then both single-trait and multi-trait genomic predictions led to significant improvements when some genotypes were fully or partially tested, though single-trait model got the best results	Persa et al. (2020)
Facultative wheat	Multi-trait model increased genetic gains vis-à-vis the single-trait model across environments, thus being the former an efficient strategy for selecting under variable water regimes	Guo et al. (2020)
Winter wheat	Multi-trait covariate models led to optimal predictions for grain yield under low genetic relatedness between training set and testing populations. Likewise, predictions for environments with low heritability improved after adding multiple traits in the model	Lozada and Cater (2019)
Wheat	Including correlated traits in both training and breeding populations may allow replacing the phenotyping of labor-intensive and costly-testing traits	Lado et al. (2018)
	Genomic prediction models for selection based on breeding values appear to be very suitable for predicting line performance in new environments if phenotypic data are available for a subset of the total testing environments	Ward et al. (2019)

### Alleviating tradeoffs in crop breeding

MicroRNA (mRNA), a small noncoding RNA molecule of 20–24 nucleotides, plays a critical role in plants stress tolerance and growth, development, and reproduction (GDR). Several drought responsive mRNAs have been discovered both in cereals and grain legumes. Changes in mRNAs under stress correlate well with increased expression of stress related genes in tolerant germplasms (Dwivedi et al., 2018). mRNAs involved in stress tolerance exerts an unwanted pleiotropic effect on GDR (Tang and Chu, 2017). A time course genotype- and stage-dependent study of mRNAs responses to a long-term drought in rice unfolded 354 drought responsive mRNAs (DRMs), grouped into five clusters, formed complex regulatory network and significant impact on the rice transcriptome. Two hundred eleven DRMs were predicted to be associated with drought tolerance (DT) or GDR. Thirty mRNAs were inversely correlated (i.e., negative tradeoff) with DT and GDR, while 21 were positively associated (i.e., no tradeoff) with DT and GDR. A better understanding of mRNAs roles in DT and GDR may therefore facilitate avoiding mRNAs with inverse relationships on DT and GDR in crop breeding (Xia et al., 2020).

*Ideal Plant architecture 1* (*IPA1*) controls both defense and productivity in rice. Wang et al. (2018) showed that reversible phosphorylation of *IPA1* allows plants to defend against fungal attack when needed but reallocate resources within days back to growth, sustaining both pathogen defense and crop yields. Moreover, it was shown that modification in the expression of *IPA1* increases resistance to bacterial blight (*Xanthomonas oryzae* pv. oryzae) and substantially increases yield in rice. How does it happen? The downregulation of miR-156 and overexpression of *IPA1* and *OsSPL7*, the two target genes of miR-156, enhances disease resistance but reduces rice yield. Gibberellin signaling partially contributed to adverse developmental defects in the *IPA1* overexpressors. However, transgenic plants expressing *IPA1* with pathogen inducible promoter showed enhanced host plant resistance and yield. Thus, miR-156-*IPA1*, a novel regulator of the crosstalk between growth and defense achieves both high disease resistance and high yield in rice (Liu et al., 2019b).

*DEHYDRATION-RESPONSIVE ELEMENT-BINDING PROTEIN 1A* (*DREB1A*) confers abiotic stress tolerance but reduces plant growth, development, and reproduction (Kasuga et al., 1999; Morran et al., 2011). However, this adverse impact on productivity could be minimized by co-expressing the growth-enhancing genes such as *GA5* and *PIF4* whose expression is repressed in abiotic stressed environments. Using *GA5* and *PIF4* for growth improvement, Kudo et al. (2019) noted enhanced biomass production in the *GA5 DREB1A* and *PIF4 DREB1A* compared to *DREB1A* overexpressors. *GA5 DREB1A* overexpressors maintained high levels of drought stress tolerance while *PIF4 DREB1A* overexpressors lower level of stress tolerance than the *DREB1A* overexpressors due to repressed expression of *DREB1A*. *GA5 DREB1A* overexpressors additively affected primary metabolism, gene expression, and plant hormone profiles in the plants, indicating that inherent tradeoff between growth and drought stress tolerance may be minimized by carefully selected genes for stacking.

Abscisic acid (ABA) is a key stress hormone regulating abiotic stress tolerance via signal transduction (Song et al., 2016; Yoshida et al., 2014). ABA-induced transcription repressors (AITRs) are a novel family of transcription factors conserved in angiosperm, with *Arabidopsis* containing six such genes (*AITR1-6*) encoding AITRs, and functions as negative regulators in regulating ABA signaling and abiotic stress tolerance (Tian et al., 2017). Knocking down of *AITR* family genes in *Arabidopsis* by CRISPR/Cas9 genome editing system (Knott and Doudna, 2018) enhanced abiotic stress tolerance without fitness costs, for example, knocking down of *AITR3* and *AITR4* simultaneously in the *aitr256* triple and *aitr1256* quadruple mutants respectively, reduced sensitivities to ABA, and enhanced tolerance to drought and salt without adversely impacting plant growth and development. Neither the plant growth and development nor plant response to pathogen infection was affected in *aitr123456* sextuple mutants. Thus, *AITRs* are an excellent target for improving abiotic stress tolerance in plants including crops (Chen et al., 2021). Jasmonate (JA) regulates growth- and defense-related processes in plants by triggering genome-wide transcriptional changes to optimize plant fitness in hostile environment (Guo et al., 2018a,c; Major et al., 2017). JA-inducible bHLH transcription factors in tomato (*Solanum lycopersicum*), MYC2-TARGETED BHLH1 (MTB1), MTB2, and MTB3, function as negative regulators of JA mediated biotic stress response, and the CRISPR/Cas9 generated mutants showed enhanced resistance to insect attack (*Helicoverpa armigera*) without fitness costs (Liu et al., 2019a). It opens new avenues to exploit *MTB* genes in crop breeding.

In addition, genes that modulate tradeoffs (Table 2) may be deployed in crop breeding. Next question is what breeding strategy one follows to make use of these novel discoveries in crop breeding? A combination of genomic selection and optimal cross selection to recurrently improve genetic resources with elite lines (Allier et al., 2020) or a multi-objective optimized breeding schemes within the phenotypic or genomic breeding frameworks (Akdemir et al., 2019) and comparisons with the standard multi-trait breeding approaches may be deployed to introduce multiple traits often with antagonistic effects for developing resource-use efficient and nutritionally enhanced crop genetic resources with enhanced adaptation.

## Conclusions

Just as with physics, for every ‘plant action’ there is a reaction and often this represents a trade-off. For example, that between additional growth of organs versus storage of photo-assimilates, between seed size and number, root:shoot, etc. The challenges to crop breeding represented by the trade-offs discussed in this review are manifold. On the one hand, increasing both seed size and seed number may boost yield potential (Calderini et al., 2021) but under harsh conditions, a tradeoff between the two is essential in ensuring seed of a commercially useful weight and viability, albeit at lower numbers. Similarly, storage of photo-assimilates in the stems of cereals is essential to achieve seed filling when unfavorable conditions during grain filling–drought, defoliation due to disease or pests, etc.–inhibit current photo-assimilation. However, in another situation or genetic background, the same assimilates might have been used in root growth to explore the subsoil for water (Lopes and Reynolds, 2010) or to grow new photosynthetic tissue to compensate for loss of functional leaf area. If an environment is well characterized, such tradeoffs can in theory be managed genetically. Nonetheless, no two farmers’ fields or growing seasons are ever exactly the same. We rely on the plasticity of our best cultivars to drive the necessary trade-offs that maximize yield within a target population of environments, and when they do not, new crossing strategies must be designed.

The road ahead will require crop science to better understand the genetic and eco-physiological bases of key adaptive tradeoffs so as to manage them within the parameters defined by specific target environments. In doing so, we must keep in mind that maximizing crop productivity is not necessarily consistent with the long-term evolutionary history of a crop species or its progenitors (as discussed previously for delayed silking under drought in maize, for example). Genetic improvement will likely require specific knowledge gaps to be filled, an area recently reviewed by crop scientists from industry and academia. The consensus on some major bottlenecks to understanding (i.e., root growth, hormone cross-talk, maintenance respiration, and source-sink balance) would if addressed allow more comprehensive crop models and generate a number of new opportunities in relation to optimizing tradeoffs, including better targeted exploration of genetic resources, more strategic crossing and progeny selection and novel crop management interventions (Reynolds et al., 2021).
